# A Review: Recent Advances of Piezoelectric Photocatalysis in the Environmental Fields

**DOI:** 10.3390/nano14201641

**Published:** 2024-10-12

**Authors:** Zhengjie Ye, Ru Zheng, Shuangjun Li, Qing Wang, Rui Zhang, Chenjing Yu, Jia Lei, Xiaoyan Liu, Dieqing Zhang

**Affiliations:** The Education Ministry Key Lab of Resource Chemistry, Joint International Research Laboratory of Resource Chemistry, Shanghai Key Laboratory of Rare Earth Functional Materials, Shanghai Frontiers Science Center of Biomimetic Catalysis, College of Chemistry and Materials Science, Shanghai Normal University, Shanghai 200234, China; 1000527217@smail.shnu.edu.cn (Z.Y.); zhengruhr1988@shnu.edu.cn (R.Z.); sjlqyz@163.com (S.L.); wq978961550@163.com (Q.W.); 1000528894@smail.shnu.edu.cn (R.Z.); 1000549596@smail.shnu.edu.cn (C.Y.); 1000526919@smail.shnu.edu.cn (J.L.)

**Keywords:** piezoelectric photocatalysis, piezoelectric effect, photocatalysis, environmental protection

## Abstract

Piezoelectric photocatalysis can effectively suppress the recombination of electron holes during the course of photocatalysis, which has been widely applied in environmental and energy catalysis. Its advantage is that when the piezoelectric effect happens, a built-in electric field is formed inside the catalyst, which improves the separation efficiency of photogenerated charge carriers and obtains more excellent photocatalytic performance. The efficient conversion of mechanical energy to chemical energy can be realized through the synergistic effect of the piezoelectric effect, and photocatalysis is greatly significant in solving the energy crisis and providing environmental protection. Therefore, we organized a more complete review to better understand the mechanism and system of piezoelectric photocatalysis. We briefly introduce the principle of the piezoelectric effect, the existing types of piezoelectric photocatalysts, the practical application scenarios, and the future challenges and feasible methods to improve catalytic efficiency. The purpose of this review is to help us broaden the idea of designing piezoelectric photocatalysts, clarify the future research direction, and put it into more fields of environmental protection and energy reuse.

## 1. Introduction

With the development of industry and the innovation of science and technology, environmental protection and energy recycling have gradually attracted the attention of domestic and international communities [1]. Therefore, finding a sustainable and clean energy source is the key to solving these problems. As a new type of environmental protection, safe and pollution-free energy from solar energy, which transforms the sun’s light energy into other forms, such as heat energy, electric energy, and chemical energy, has once again aroused people’s attention [2]. As early as 1972, Fujishima and Honda first reported the phenomenon that TiO_2_ single crystal electrode photocatalyzed water produced hydrogen, which opened the prelude to photocatalysis [3]. Photocatalysis, as an advanced oxidation technology, converts clean, non-polluting, and renewable solar energy in nature into chemical energy and is considered a truly environmentally friendly technology. It can be applied in the field of not only the degradation of environmental pollution, including organic and inorganic pollutants, but also in the field of energy regeneration, such as the conversion of solar energy into green, storable, clean energy hydrogen energy [4,5,6]. However, there are also many difficulties in the process of photocatalysis, such as the low efficiency of light energy utilization and the high recombination rate of light-generated electron holes. Conventional photocatalysts have found it difficult to break through the limitations of their own carrier transfer difference.

**Figure 1:**
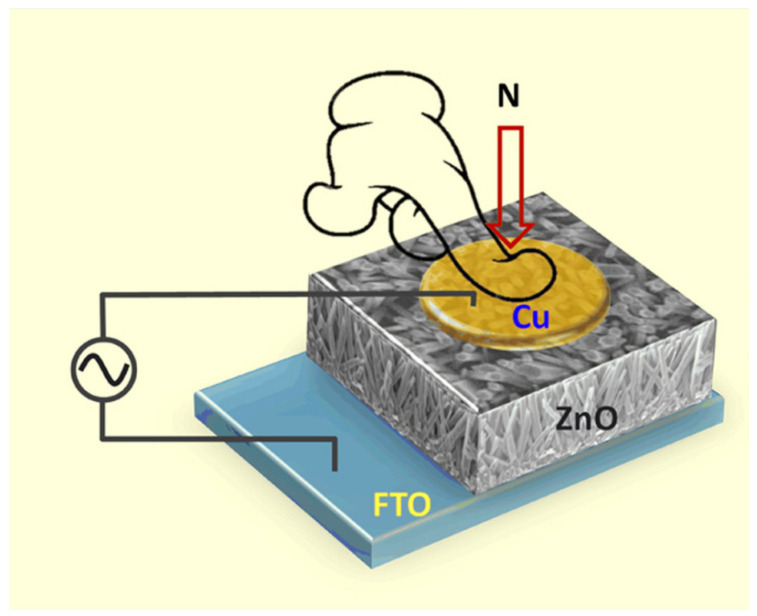
Schematic diagram of piezoelectric effect. Reproduced from Ref. [27]. Copyright, 2017. Elsevier.

Piezoelectric catalysis has received much attention as a new advanced oxidation technology. This concept was first discovered in Jacks’ laboratory by Pierre P Curie and Jacks J Curie in 1880 [7]. At first, the piezoelectric effect was not valued until the piezoelectric materials were widely used in industry, energy, military, biological applications, and engineering [8,9]. The piezoeletric effect is shown in Figure 1. For example, a piezoelectric material may be used as a sensor to detect pressure, force, or strain by measuring the charge or voltage change that it produces. This sensor is widely used in the mechanical field, medical equipment, the automotive industry, and so on [10,11,12]. Piezoelectric materials differ from other common piezoresistive materials. They can detect electrical signals generated on the surface by applying a force that results in the polarization of an internal charge. In contrast, piezoresistive materials produce a change in resistance when a force is applied. We sense the pressure on the sensor by observing the change in resistance [13]. Piezoelectric materials do not require an external power supply. For example, piezoelectric ceramics are a type of special piezoelectric material that can undergo mechanical deformation under the action of the electric field or can produce an electrical signal when an external force is applied. Therefore, piezoelectric ceramics are widely used in ultrasonic generators, ultrasonic sensors, piezoelectric ceramic motors, and other fields [14,15,16]. Piezoelectric materials can generate potential by external forces. This feature allows piezoelectric materials to be used in energy collection and storage devices, making self-powered sensors, smartphone charging devices, and the like [17]. 

Piezoelectric photocatalysis is a new catalytic technology combining the piezoelectric effect and photocatalytic effect. Under external stress, polarization and a built-in electric field are constructed, which helps to improve the efficiency of light absorption and charge carrier separation [18,19]. Meanwhile, light induces the catalysts to promote a reaction [20]. Piezoelectric photocatalysis technology also has some unique advantages. First, by regulating the applied stress of piezoelectric materials, the rate and effect of catalytic reactions can be precisely controlled [21]. This tunability gives piezoelectric photocatalysis great potential for optimizing reaction conditions. Second, the piezoelectric photocatalytic technology can synergistically realize the energy conversion of light and mechanical energy, thus improving energy utilization efficiency. By combining piezoelectric materials with renewable energy sources or mechanical motion, highly efficient energy conversion and utilization can be achieved. Thus, piezoelectric photocatalysis has also been applied in the field of environmental governance, which can be used for the photodegradation of pollutants and the photocatalytic conversion of carbon dioxide into useful fuels [22,23,24,25,26]. This technology can not only reduce the discharge of environmental pollutants but also convert waste into renewable resources. However, piezoelectric photocatalysis faces some challenges in practical applications, such as material stability, reaction efficiency, and cost. In order to overcome the above-mentioned difficulties, many efforts have been devoted to piezoelectric photocatalysis in the field of environmental protection. In this review, we summarize the recent advances in the following four aspects of piezoelectric photocatalysis: (1) photocatalytic principle and piezoelectric photocatalytic principle, (2) the type of piezoelectric photocatalysts, (3) the practical application of piezoelectric photocatalysis, and (4) the design and challenges of piezoelectric photocatalysis in the future.

## 2. Principles of Photocatalysis, Piezoelectric Catalysis and Piezoelectric Photocatalysis 

### 2.1. Principle of Photocatalysis

Photocatalysis is a process based on the interaction of light with matter, whereby light excites electrons and holes in a material to prompt a chemical reaction. The mechanism of a photocatalytic reaction involves several processes, including light excitation, carrier generation and transport, and surface reaction. First, light energy excites the electron and hole pairs in the semiconductors to form an excited state. Then, the charge carriers in the excited state diffuse and are transported in the material to reach the reaction interface. Finally, the carriers participate in the surface reaction and have a chemical reaction with the target material, thus catalyzing the target reaction. Specifically, the electrons and holes generated in the process of the light excitation of compound semiconductors, such as titanium dioxide, are used to participate in the redox reaction. When light with an energy greater than or equal to the energy gap is irradiated onto a semiconductor nanoparticle, the electrons in its valence band will be excited to jump to the conduction band, leaving relatively stable holes in the valence band, thus forming an electron-hole pair. Due to the presence of a large number of defects and dangling bonds in nanomaterials, these defects and dangling bonds can capture electrons or holes and prevent the recombination of electrons and holes. These captured electrons and holes diffuse to the surface of the particles, respectively, resulting in a strong redox potential. Photocatalytic technology has a wide range of applications in the fields of energy conversion, environmental treatment, organic synthesis, etc. It has been applied in the fields of NO_3_^-^ reduction, N_2_ fixation, NO oxidation, NO reduction to produce NH_3_, CO_2_ reduction, H_2_O_2_ production, and so on [28,29,30,31].

### 2.2. Principle of Piezoelectric Effect and Piezoelectric Catalysis

**Figure 2:**
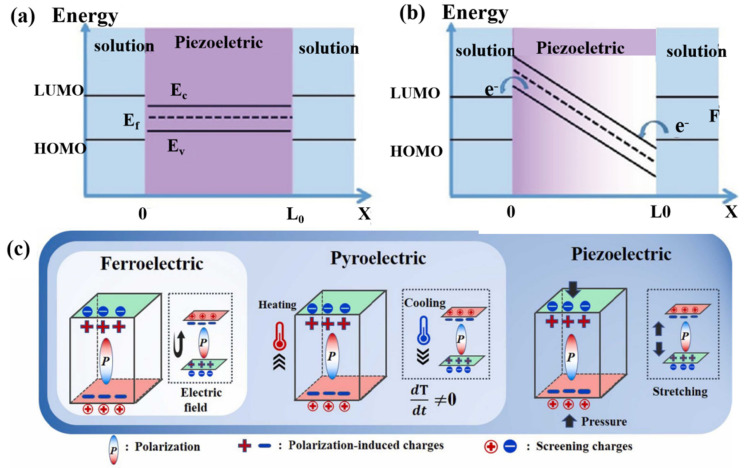
Band diagram of (**a**) unstrained and (**b**) strained piezoelectric effect. Reproduced from Ref. [35] with permission. Copyright, 2020. Wiley. (**c**) The mechanisms of ferroelectricity, pyroelectricity, and piezoelectricity. Reproduced from Ref. [36] with permission. Copyrght, 2024. Elsevier.

The piezoelectric effect is a phenomenon of interaction between mechanical and electrical energy discovered by the French Curie brothers in 1880 by studying the symmetry of crystals. It produces an electric charge when you apply pressure, and it deforms when you apply an externally built electric field, called the piezoelectric effect. In the piezoelectric body, when subjected to external stresses that occur in the electrode and which lead to the piezoelectric body at both ends of the surface being of the oppositely bound charge, the charge density is proportional to the size of the stress, also known as the positive piezoelectric effect [32,33]. Back in 2006, Zhonglin Wang was the inventor of the nanogenerator and first realized the conversion of mechanical energy into electrical energy at the nanoscale. He converted microscopic electrons into macroscopic electrical energy by cyclically squeezing and releasing the material to collect the surface charge [34]. Piezoelectricity has the characteristics of being able to capture ambient mechanical forces, having an adjustable polarized electric field, and indicating an enriched charge, so it is feasible to combine the piezoelectric effect with catalytic reactions for application in the fields of energy crisis and environmental protection. Piezoelectric catalysis involves the use of the positive piezoelectric effect to generate an electric charge, with the separation of electrons e^-^ and holes h^+^, which interact with O_2_ and OH^-^ in the system to produce hydroxyl radicals ·OH and superoxide radicals ·O_2_^−^. The performance of redox catalysis can be controlled by the piezoelectric effect; therefore, both the piezoelectric effect and piezoelectric photocatalysis can modulate charge transport behavior during charge transfer and improve the photoelectrochemical performance of piezoelectric semiconductors. According to Huang et al., the piezoelectric potential formed by the built-in electric field steals unoccupied or occupied electron energy levels in the material, and it lowers the conduction band (CB) of the solution to below the highest occupied molecular orbital (HOMO) [35]. As a result, the electrons are transferred from homo to CB, and on the other side, the electrons leave the valence band (VB) and transfer to the lowest unoccupied molecular orbital (LOMO), as shown in Figure 2a,b. In crystallography, there exists the concept of a 32-point group, 11 of which are center-symmetric and not piezoelectric. The rest of the point groups are piezoelectric except for the special 432-point group (which has no center of symmetry but is highly symmetric in structure). Half of the remaining 20-point groups with piezoelectricity have a unique polar axis, whose spontaneous polarization then changes with temperature, i.e., pyroelectric materials. Among these, ferroelectrics are crystals present in pyroelectric crystals that are capable of spontaneous polarization and can be inverted by an applied electric field. Both pyroelectrics and ferroelectrics are piezoelectrics. The difference between them is that whereas piezoelectrics require external stress to induce polarization, pyroelectrics induce polarization through temperature changes (warming or cooling); ferroelectrics can be polarized on their own, and the direction of polarization can be reversed in the presence of an applied electric field. A detailed diagram is shown in Figure 2c [36]. Among them, ferroelectric materials also have a special temperature range—also known as Curie temperature. Below the Curie temperature, the ferroelectric effect is present, and conversely, the ferroelectric effect transforms into a cis electrode with a ferroelectric effect. Near the Curie temperature, ferroelectric materials tend to show good activity. Therefore, the Curie temperature is an important parameter of ferroelectric materials, which not only determines the temperature range of ferroelectricity in the material but also affects the performance of the material in practical applications [37,38].

### 2.3. Principle of Piezoelectric Photocatalysis

**Figure 3:**
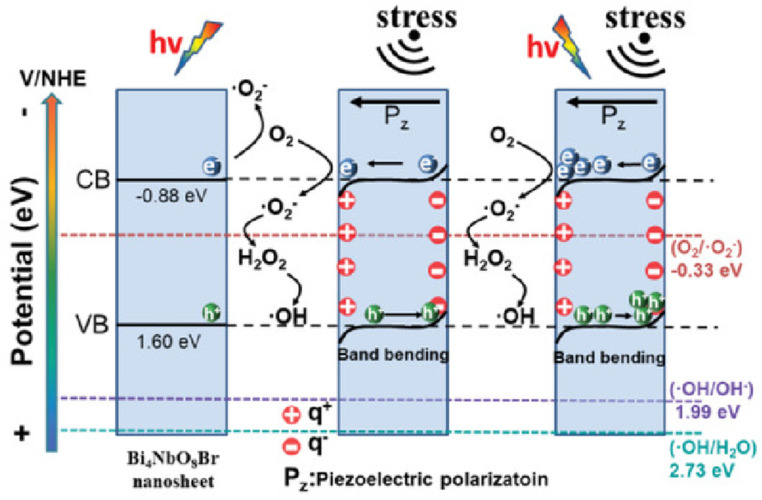
Band bending of Bi_4_NbO_8_Br in different conditions. Reproduced from Ref. [35] with permission. Copyright, 2020. Wiley.

Piezoelectric photocatalysis means that the piezoelectric photocatalyst receives light irradiation while being subjected to the external stress given to the catalyst, which deforms the surface of the catalyst. This deformation forms a built-in electric field, which accelerates carrier separation and effectively inhibits the complexation of photogenerated carriers, thus improving the photocatalytic performance of the catalyst. In piezoelectric photocatalysis, the deformation of the piezoelectric material due to mechanical stress generates an internal voltage that forms a built-in electric field, which facilitates the separation of photogenerated carriers, and thus improves the semiconductor’s photocatalytic performance. The main ways to obtain the driving force of the piezoelectric effect are mechanical vibration, ultrasound, thermal stress, bending stress, and air friction [39,40,41,42]. When the surface is polarized, the surface charge is affected and shifts, thus affecting the release and adsorption of the charge. Therefore, we can change the strength and direction of the electric field by adjusting the direction and strength of the external stress so that we can realize the precise control of the charge and improve our catalytic efficiency. Piezoelectric materials have a non-centrosymmetric structure and undergo polarization due to the relative displacement of positive and negative charges under mechanical strain. Interfacial redox reactions occur when the surface of this polarized material has sufficiently high Gibbs free energy. It is induced by piezoelectric polarization, and different faces of the piezoelectric material have different Gibbs free energies. For crystals with centrosymmetric structures, they can also be piezoelectric by breaking the inversion of symmetry through the adsorption of heteroatoms, the introduction of specific in-plane defects, and non-uniform deformations of the strain gradient induced by the electrodeposition of the internal deflection [43,44,45]. Energy band bending caused by positive and negative polarization charges in piezoelectric semiconductors alters the energies of photogenerated electrons and holes located in the conduction and valence bands. The downward bending of the conduction band helps to enhance a reduction in photogenerated electrons, and the upward bending of the valence band helps to improve the oxidation of holes. Taking the Bi_4_NbO_8_Br catalyst as an example, the difference between photocatalysis, piezoelectric catalysis, and piezoelectric photocatalysis is shown in Figure 3, whereas in pure photocatalysis, the catalyst only produces photogenerated electron-hole pairs and in pure piezocatalysis catalysts, can only promote electron transport efficiency by inducing band bending due to the piezoelectric effect. In piezoelectric photocatalysis, the catalyst’s energy bands are bent due to the deformation produced by external stresses, creating a piezoelectric potential capable of further promoting the efficiency of charge separation. This acts synergistically with the photocatalytic generation of electron-hole pairs, promoting electron-hole pair separation through a built-in electric field and improving its catalytic performance.

## 3. Piezoelectric Photocatalytic Catalysts

### 3.1. Zinc Oxide-Based Catalysts

Zinc oxides are a thermally stable n-type semiconductor material with a wide bandgap as well as a very high exciton binding energy capable of reaching 60 meV [32,46,47]. Whether a semiconductor material is piezoelectric depends on whether the crystal structure of that material is asymmetric. Piezoelectricity is generally caused by the dipole moments of positive and negative ions inside the crystal being subjected to an external force, resulting in a disruption of the crystal’s electroneutrality, causing a residual charge to appear on the surface of the crystal in a certain direction. Zinc oxides have three crystalline structures, including hexagonal fibrous zincite, cubic sphalerite, and rarer sodium chloride octahedral. The hexagonal fibrous zincite structure is the most stable one and, therefore, the most common. In the hexagonal fibrous zincite structure, each Zn atom forms a tetrahedral structure with the four surrounding O atoms, and each O atom also forms a tetrahedral structure with the four Zn atoms. This arrangement gives ZnO two polar surfaces. The crystal structure of ZnO with tetra-coordination leads to the lack of central symmetry of ZnO itself, so it has a piezoelectric effect. In semiconductor materials, Zn and O are mostly combined with ionic bonds, which is also one of the reasons for their high piezoelectric performance. When an external force is applied to the crystal, the previously overlapping anionic and cationic centers are shifted relative to each other, making the dipole moment non-zero and creating a macroscopic potential difference. ZnO has a strong electromechanical coupling coefficient and very stable piezoelectric properties, so it has a very wide range of applications in piezoelectric sensing, piezoelectric converters, and piezoelectric catalysis [32,34,43,48,49].

**Figure 4:**
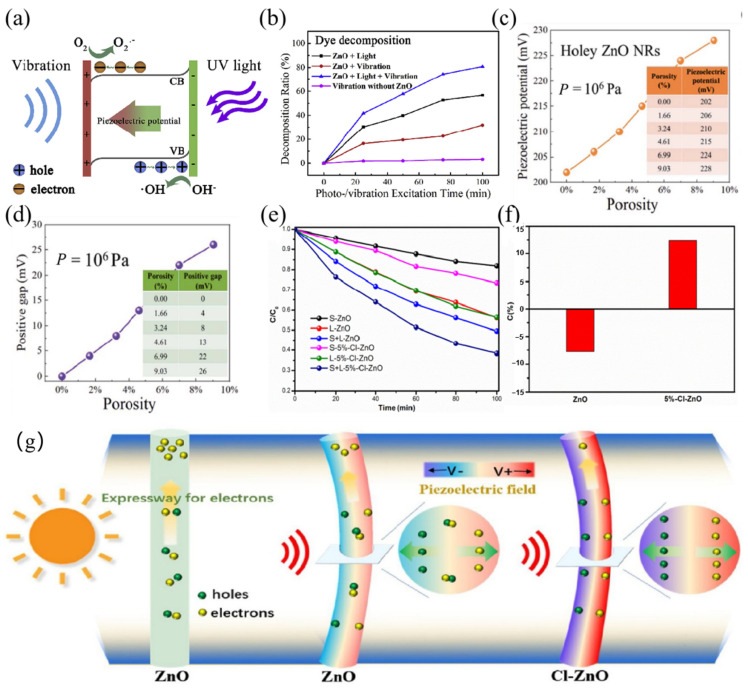
(**a**) The schematic illustration of dye decomposition by bi-harvesting photo vibration energy. (**b**) Comparison of the decomposition ratios of the AO7 solution under different external conditions. Reproduced from Ref. [52] with permission. Copyright, 2019. Elsevier. (**c**) Plots of piezoelectric potential of holey ZnO NRs versus porosity. (**d**) Plots of the gap between piezoelectric potential of ZnO NRs and holey ZnO NRs versus porosity. Reproduced from Ref. [53] with permission. Copyright, 2023. (**e**) Catalytic degradation of 2-NAP by ZnO and 5%-Cl-ZnO under light irradiation (L), ultrasound (S), and light irradiation and ultrasound simultaneously (S+L). (**f**) The coupling constant (C) of the coupling effect of piezo-photocatalysis. (**g**) Schematic illustration for the catalytic mechanism of ZnO and Cl-ZnO under different external conditions. Reproduced from Ref. [54] with permission. Copyright, 2020. Elsevier.

In recent years, research on ZnO piezoelectric catalysis has attracted intensive attention, focusing on the piezoelectric photocatalytic of ZnO. Forming composite catalysts with ZnO and other piezoelectric catalysts or photocatalysts to enhance the piezoelectric properties of ZnO can improve the degradation rate of pollutants as well as increase the yield of hydrogen production [50,51]. In 2019, Liu et al. synthesized ZnO nanorods using a hydrothermal method and successfully achieved the dual harvesting of energy from light and vibration and the decomposition of dye acid orange 7 by piezoelectric photocatalysis, which led to a decomposition rate of 80.8% in the simultaneous presence of both light and vibration, which is much higher than that of photocatalysis alone at 56.7% and the piezoelectric catalyst at 31.8% [52]. This work focuses on the bending of ZnO nanorods by vibrational energy, which induces the generation of piezoelectric potentials and massive charge generation through the piezoelectric effect. Liu et al. applied stress to the nanomaterials through the liquid ultrasonic cavitation effect, and the piezoelectric potential could be modulated by adjusting the frequency of vibrational energy, which may have led to the accelerated rate of the decomposition of acid orange 7, but it did not actually affect the final decomposition. In order to simulate the degradation of dye contaminants, the ZnO catalyst was placed in an acid orange 7 solution to reach adsorption equilibrium and was then placed in the center of a vibration source and under a stable room temperature UV light source. When the ZnO nanorods collected light energy, the electrons transferred to the conduction band (CB), and holes were generated in the valence band (VB). The vibration-generated piezoelectric potential and a large number of charges further enabled the separation of photogenerated electrons and holes, which effectively degraded the dye pollutant acid orange 7 as shown in Figure 4a,b. Li et al. prepared porous ZnO nanorods from formaldehyde using a hydrothermal method [53]. Since formaldehyde occupies part of the growth space of ZnO and leaves holes inside the nanorods after annealing, these built-in nanopores can induce the formation of tensile stresses and increase their piezoelectric potential, which promotes its piezoelectric properties. The ZnO nanorods with holes also have a higher piezoelectric potential than the ZnO nanorods without holes, which is more conducive to improving the piezoelectric catalytic efficiency, as shown in Figure 4c,d.

Although single ZnO can promote its photocatalytic efficiency by virtue of its piezoelectricity, it still has the disadvantage of insufficient piezoelectric efficiency. Several strategies have also been reported to enhance piezoelectric performance by modification, such as doping, constructing heterojunctions to enhance the carrier transfer rate, etc. Liu et al. achieved piezoelectric tuning and energy harvesting by doping Cl into ZnO nanorods via solvothermal synthesis, improving the coupling effect of piezoelectric photocatalytic dye decomposition [54]. They found an improved method of fabricating nanogenerators using piezoelectric materials that can modulate the piezoelectric properties of ZnO. Doping Cl can cause significant lattice expansion, leading to the asymmetric structure of the crystals without destroying the integrity of the crystals. In Figure 4e,f, the test for the degradation of rhodamine B was used as a probe reaction to observe the normalized concentration change (C/C_0_) of the RhB solution during photodegradation under the combined irradiation of ultrasound and light and calculate coupling constants for piezoelectric photocatalytic coupling effects. According to their results, pure ZnO showed inferior performances under both photocatalysis alone and piezoelectric catalysis using ultrasound generation. Moreover, the piezoelectric photocatalytic performance of Cl-doped ZnO was even more remarkable, which is due to the fact that the axial direction of ZnO NRs is a fast channel for electron migration, and the rapidly transferred electrons cannot be consumed in a timely manner, resulting in the accumulation of electrons at the end of the NRs. However, Cl-doped ZnO provides a larger radial piezoelectric field, allowing more carriers to migrate to the sides, as shown in Figure 4g. This work not only demonstrates the high correlation between piezoelectricity and the coupling effect of piezoelectric photocatalysis but also provides new insights into the modulation of piezoelectricity in order to improve catalytic activity.

### 3.2. ABO_3_ Catalysts

As a conventional piezoelectric semiconductor material, chalcogenide-type materials, along with ZnO, carbon nitride, and bismuth-based metal oxides, have been widely used in energy conversion and environmental remediation. Chalcogenide-type materials have been previously used as solar cells and nanogenerators for research and applications, and their crystals have a non-centrosymmetric structure, which leads to electrodeposition when subjected to mechanical stress [55]. They usually exhibit the ABO_3_ configuration, where A represents the larger cation, B represents the smaller cation, and O represents the oxygen ion. In this structure, the B-site ions (e.g., titanium ions) form an octahedral coordination with the surrounding oxygen ions, and when the position of the B-site ions in the crystal lattice is slightly shifted, this results in the non-overlap of positive and negative centers of charge in the crystal cell, which leads to an electric dipole moment. Common chalcogenide materials include BaTiO_3_, SrTiO_3_, and PbTiO_3_ [23,56,57]. When external mechanical stresses are applied to the chalcogenide material, the B-site ions in the crystal cell further deviate from the central position, enhancing the degree of electrodeposition of the material, which, in turn, creates voltage at the two ends of the material, creating a piezoelectric effect. This phenomenon allows the chalcogenide material to convert mechanical energy into electrical energy and vice versa.

**Figure 5:**
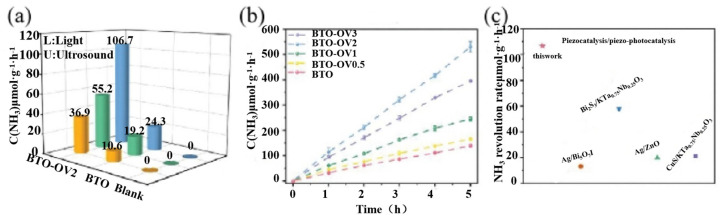
(**a**) NH_4_^+^ yields of blank, BTO, and BTO-OV2 under L+U irradiation using sodium sulfide/sodium sulfite as a sacrificial agent. (**b**) Comparison of NH_3_ production rate of BTO-OV2 with a reported piezoelectric/piezoelectric-photocatalytic nitrogen fixation system. (**c**) NH_4_^+^ yields of BTO-OVX under L+U irradiation in pure water. Reproduced from Ref. [23] with permission. Copyright, 2023. Wiley.

Chalcogenides exhibit high dielectric constants and piezoelectric coefficients due to their special phase structure and the dispersion of positive and negative centers, which not only give them excellent piezoelectric properties but also a variety of physicochemical properties, such as ferroelectricity, multiferroics, dielectricity, photovoltaics, catalysis, and so on. There have been many research reports related to ATiO_3_ in recent years, demonstrating its excellent piezoelectric photocatalytic properties. In 2023, Dai et al. introduced controllable OVs into barium titanate and systematically revealed the synergistic mechanism of oxygen vacancy defects and the piezoelectric polarization field in piezoelectric-photocatalytic nitrogen fixation, and the piezoelectric-photocatalytic NH_3_ yield of the optimal sample was 106.7 μmol g^−1^ h^−1^, as shown in Figure 5a [23]. The performance of the sample was higher than that of the currently reported piezoelectric/piezoelectric photocatalysts in Figure 5c. The introduction of oxygen defects changes the local dipole state of BaTiO_3_ and enhances the piezoelectric polarization of BaTiO_3_, which promotes photogenerated carrier separation more effectively. BTO, with different defect concentrations, demonstrated different NH_3_ yields, indicating that the introduction of moderate concentrations of OV can enhance the piezoelectric performance of BTO, as shown in Figure 5b; the piezoelectric field can modulate the d-band center of the Ti3^+^ catalytic site and promote the activation and dissociation of N_2_, which greatly reduces the reaction barriers of the decisive velocity step. BTO, with different defect concentrations, demonstrated different NH_3_ yields, indicating that the introduction of moderate concentrations of OV can enhance the piezoelectric performance of BTO and the piezoelectric field can modulate the d-band centers of Ti3^+^ catalytic sites and promote the activation and dissociation of N_2_, which greatly reduces the reaction barriers of the decisive velocity step.

**Figure 6:**
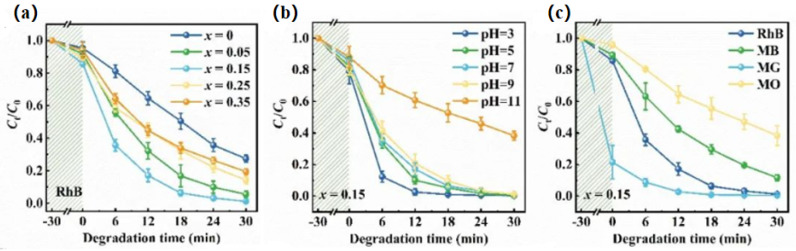
(**a**) Degradation curves of RhB by Ba_1-x_Sr_x_TiO_3_ under UV light and ultrasonic vibration as a function of time (**b**) Degradation efficiency of Ba_0.85_Sr_0.15_TiO_3_ at different pH values (**c**) for different dyes. Reproduced from Ref. [58] with permission. Copyright, 2023. Elsevier.

Wu et al. proposed a facile strategy to modify BaTiO_3_ by Sr^2+^ doping, and Ba_1-x_Sr_x_TiO_3_ nanopowders were prepared by the sol–gel method [58]. It was demonstrated that Sr^2+^ doping could improve the catalytic efficiency of BaTiO_3_ in three ways, namely, changing the energy band gap, regulating the grain size, and lowering the Curie temperature. The optimal catalytic performance for the degradation of rhodamine B (RhB) via piezoelectric-optical synergism was achieved at x = 0.15, with a degradation rate of 92.66% in 18 min and a reaction rate constant of 14.26 × 10^−2^ min^−1^, which exceeds that of most BaTiO_3_^−^ based materials, as shown in Figure 6a. This material has demonstrated adaptability to different environments and applications in practical applications. Practical applications show the ability to adapt to the diverse requirements of different environmental pollutants, as shown in Figure 6b,c.

### 3.3. g-C_3_N_4_-Based Catalysts

**Figure 7:**
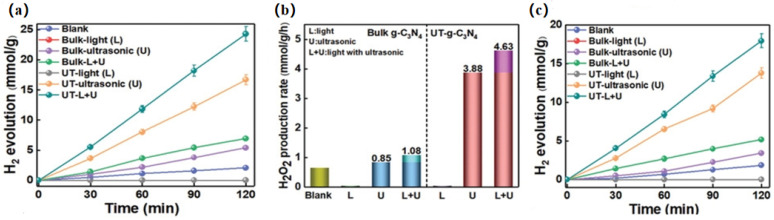
(**a**) The H_2_ yields and (**b**) production rates of bulk g-C_3_N_4_ and UT-g-C_3_N_4_ under light, ultrasound, and simultaneous light and ultrasonic irradiation in glucose solution. (**c**) The H_2_O_2_ production rate of bulk g-C_3_N_4_ and UT-g-C_3_N_4_ under different conditions. The H_2_ yields and production rates of bulk g-C_3_N_4_ and UT-g-C_3_N_4_ under light, ultrasound, and simultaneous light and ultrasonic irradiation in pure water. Reproduced from Ref. [61] with permission. Copyright, 2021. Wiley.

Graphitic carbon nitride has been demonstrated as a potential photocatalyst for environmental purification, but its photocatalytic efficiency is low due to its high photogenerated carrier complexation rate. Introducing built-in electric fields into photocatalysts has become a major research hotspot [6,19,44,45,59,60]. Since the crystal structure of bulk graphitic carbon nitride is centrosymmetric, graphitic phase carbon nitride itself is not piezoelectric, but through the triazine layer of modified graphitic carbon nitride, the triangular nanopores produce the strong bending of electrical properties in the triazine plane, which leads to the distortion of the laminar plane of carbon nitride, and leads to the enhancement of the polarization field, resulting in a piezoelectric response. Therefore, increasing its polarity can increase piezoelectricity, which is seen as a viable strategy. In 2021, Hu et al. prepared atomically thick g-C_3_N_4_ nanosheets of non-piezoelectric bulk phase g-C_3_N_4_ into two-dimensional nanosheets using a combined stripping technique [61]. Due to the symmetry breaking of g-C_3_N_4_ after turning into a two-dimensional material, the strong piezoelectricity was demonstrated by the in-plane polarization generated by the consistent arrangement of the internal polar heptazine ring units and the flexoelectric effect together. UT-g-C_3_N_4_ and bulk g-C_3_N_4_ hardly produce H_2_ under light irradiation. Hydrogen was produced from both samples under ultrasonic irradiation, with hydrogen precipitation rates of 8.35 and 2.69 mmol g^−1^·h^−1^ for UT-g-C_3_N_4_ and bulk g-C_3_N_4_, respectively. The higher H_2_ yield may be due to its greater piezoelectricity and more efficient vibrational energy harvesting, as shown in Figure 7a. In addition, the main oxide it produces is the highly valuable hydrogen peroxide, as shown in Figure 7b. Moreover, pure water cracking experiments were carried out on bulk g-C_3_N_4_ and UT-g-C_3_N_4_ under different conditions, and UT-g-C_3_N_4_ showed the highest hydrogen production rate under light and ultrasound, which was three times higher than that of native g-C_3_N_4_ and more than 30% higher than that under ultrasound, as shown in Figure 7c. This study provides a new and effective way to utilize mechanical and solar energy for hydrogen production and, at the same time, expands the applications of g-C_3_N_4_, laying the foundation for the development of g-C_3_N_4_-based polar semiconductors as high-performance piezoelectric catalysts.

**Figure 8:**
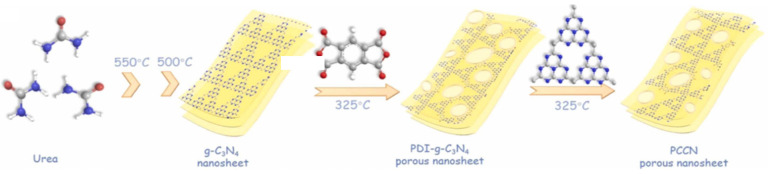
The synthetic route of the catalysts. Reproduced from Ref. [45] with permission. Copyright, 2021. Elsevier.

In the same year, Li et al. prepared g-C_3_N_4_/PDI-g-C_3_N_4_ (CNPC) homojunctions for the piezoelectric photocatalytic degradation of atrazine using a thermal condensation method [45]. The introduction of PDI enhances π-π interactions to promote electron migration and distorts the g-C_3_N_4_ planes into a more polar and porous structure with enhanced piezoelectricity, and the homojunctions further promote the g-C_3_N_4_/PDI-g-C_3_N_4_ interface and photoelectron transfer at the PDI-g-C_3_N_4_ interface. The specific synthesis routes are shown in Figure 8. The degradation and removal efficiency of ATZ was as high as 94%, and the homojunction generated 642.54 μM H_2_O_2_ during 60 min of piezoelectric photocatalysis.

### 3.4. BiFeO_3_-Based Catalysts

**Figure 9:**
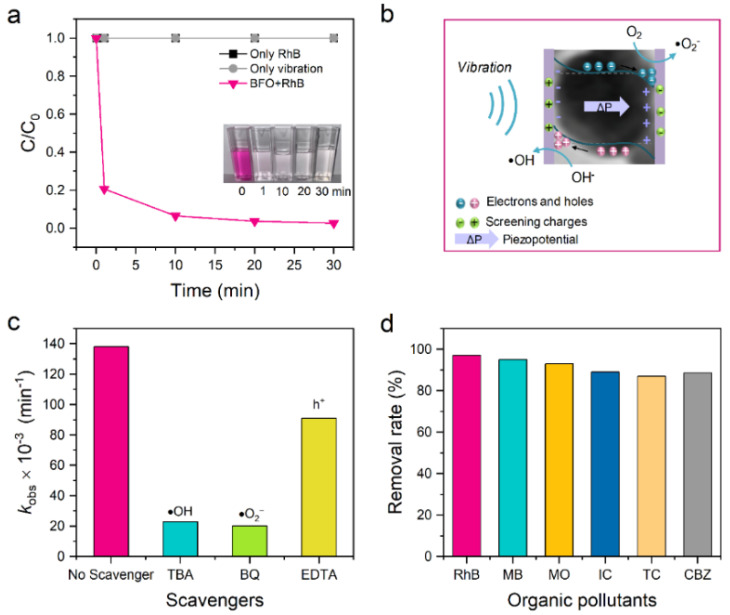
Piezocatalytic performance of BiFeO_3_ nanoparticles. (**a**) Degradation efficiency of RhB using BFO nanoparticles in the dark under ultrasonic waves (100 W, 45 kHz). The insert is a photograph of the piezocatalytic decomposition of the RhB solution in the presence of BFO nanoparticles before and after piezocatalysis. (**b**) Schematic of the BFO-nanoparticle-mediated piezocatalytic effect. (**c**) Observed constant rate of RhB degradation with and without free radical scavengers. (**d**) Removal efficiency of various dyes and pharmaceuticals using BFO nanoparticles. Reproduced from Ref. [65] with permission. Copyright, 2023. Wiley.

Ferrites are a typical ferroelectric material, a class of polar crystals with a non-centrosymmetric structure. Among these microstructures, ferroelectrics can be viewed as consisting of many small regions (domains), each with a uniform polarization direction within the domains, while adjacent domains are polarized in different directions, thus making the whole crystal non-polar from a macroscopic point of view. However, under the action of some external electric fields, polarization may be enhanced along the domains in the direction of the external field. When all the domains are along the direction of the external electric field, the whole crystal becomes a single-domain crystal, i.e., a spontaneously polarized crystal [62,63]. Ferroelectric materials are widely used in capacitors, piezoelectric sensors, storage, and microdrive applications due to their unique spontaneous polarization properties. Traditionally, ferroelectric insulators and semiconductors are two distinct materials, but in recent years, researchers have modified ferroelectric materials to have semiconductor-like properties. In 2004, Grosso et al. first proposed the potential of ferroelectric materials for photocatalytic applications, and subsequent studies have focused on the effects of specific surface areas and forbidden bandwidth on the photocatalytic activity of ferroelectric materials [64]. In recent years, it has been found that under the action of polarized electric fields, photogenerated electrons, and holes migrate in opposite directions, thus realizing the efficient separation of electron-hole pairs generated by light excitation and inhibiting the compounding of electrons and holes to a large extent, which enhances photocatalytic activity and photovoltaic conversion efficiency. In 2023, Brahim Dkhil et al. found that 60 nm-sized BFO NPs exhibited a tremendous piezocatalytic effect with record degradation rates [65]. Through a synergistic piezophotocatalytic process, the BFO nanocatalysts achieved complete degradation efficiency of model rhodamine B dye, as shown in Figure 9a, which corresponds to a kinetic rate constant of 41,750 L mol^−1^ min^−1^, while ·OH and ·O_2_^−^ are the main actives for RhB piezocatalytic degradation using BFO NPs, as shown in Figure 9b,c. And, in other pollutant tests, we found that BiFeO_3_ piezocatalytic nanoparticles are versatile for several cationic and anionic dyes, as well as pharmaceutical pollutants, with piezocatalytic decomposition rates of more than 80% within 120 min, as shown in Figure 9d. This all stems from the fact that the BFO itself has a high piezoelectric response, low dielectric constant, and low bandgap, and they also combined (PVDF-TrFE) with BFO to form a composite film, which ensured its feasibility for practical application scenarios.

### 3.5. MOF-Based Catalysts

MOF materials with high porosity, a large specific surface area, regular pore channels, and adjustable pore size are considered as a class of crystalline porous solids and metal–organic frameworks (MOFs) featuring highly tailored and customizable structures [28,66,67]. The piezoelectricity of MOF materials also mainly originate from the asymmetry of their crystal structure. MOF materials are generally formed by the self-assembly of metal ions or metal clusters and bridging organic ligands. This connection allows the formation of crystal structures with asymmetric centers. When subjected to external stresses, the relative displacement of positive and negative charge centers produces piezoelectricity. The polarity and strength of the ligand bonds also affect the charge distribution and polarization behavior, producing different piezoelectricity. In 2021, Jiang et al. synthesized UiO-66-NH_2_(Zr) and UiO-66NH_2_(Hf) for piezoelectric photocatalysis [68]. For piezoelectric photocatalysts, applying mechanical stress can produce a piezoelectric effect but, at the same time, ultrasound and mechanical stirring can intensify mass transfer during the reaction process; the UiO-66-NH_2_(Zr) and UiO-66NH_2_(Hf) have similar structures, but the activity of UiO-66-NH_2_(Hf) is about 2.2 times more active than that of UiO-66-NH_2_(Zr) in the activity test for hydrogen production, and thus, the difference in activity is identified as a function of the piezoelectric effect. This is also the first paper to clarify that the piezoelectric effect contributes to piezoelectric photocatalytic activity. In 2022, Dong et al. synthesized two different morphologies of Bi-MOFs for the degradation of rhodamine B [69]. Among them, CAU-17 (rod-shaped) exhibited a more excellent piezoelectric effect, with degradation efficiencies 3.9 times higher than those of photocatalysis and 1.5 times higher than those of piezoelectricity. Since the rod structure is more easily deformed than the sheet structure, ultrasonic vibration treatment of rod CAU-17 realizes the effective coupling of piezoelectric catalysis and photocatalysis, which is conducive to the effective separation of electrons. In 2023, Fang et al. combined CdS quantum dots with UiO-66-NH_2_(Hf) to prepare an efficient hydrogen precipitation piezoelectric photocatalyst [70]. The photogenerated charge transport distance was shortened by the unique electronic properties and quantum binding effect of CdS quantum dots as well as the built-in electric field generated by the piezoelectric polarization of UiO-66-NH_2_(Hf), which could provide a strong driving force to promote the separation of carriers. Its hydrogen precipitation rate is at 2028.48 μmol·g^−1^·h^−1^, which is four times higher than that in the absence of sonication conditions. At present, since most of the research on MOF materials regarding piezoelectricity only stays in the theoretical part of the study and does not extend to practical applications, the above literature can provide us with some inspirations and ideas for the design of MOF-related piezoelectric materials, which have a very significant potential to be linked to real-life applications.

### 3.6. Comparison of Various Piezoelectric Photocatalysts 

As shown in Table 1, we compared some reported piezoelectric photocatalysts. It was found that the morphology and structure of the catalysts are important factors for the piezoelectric effect. For example, the structure of ZnO nanorods in the table above can enhance the piezoelectric effect to further degrade liquid phase contaminants. Similarly, making the materials into two-dimensional sheets can also better induce the piezoelectric response and enhance catalytic activity. In material synthesis, constructing heterostructures in catalysts can form a built-in electric field of piezoelectricity to promote the separation and migration of photogenerated charges and, thus, improve the photoelectrocatalytic efficiency, such as PVDF-TrFE-BiFeO_3_ and UiO-66-NH_2_@CdS in the above table. The external stresses applied to the piezoelectric materials are generally induced by ultrasound. But ultrasound is costly and energy-consuming, after all. Therefore, to explore and broaden the application of other discarded and neglected stresses in nature, such as friction and vortex shear, in such experiments is one of the ideas for the development of piezoelectric photocatalysis in the future.

In line with the UN Sustainable Development Report on Energy Use and Recovery in Wastewater Treatment, the UN aims to reduce wastewater discharge and improve wastewater treatment practices to enhance water management. This will further improve the quality of water and allow people to have more access to healthy drinking water [71]. The piezoelectric photocatalysis has also been used to degrade dyes in wastewater and has shown excellent degradation results [52,54].

The piezoelectric effect can indeed convert external stress into electricity, but the amount of electricity generated is ultimately traceable, and at this stage, only piezoelectric materials have been applied to nanogenerators. But, piezoelectric photocatalytic materials can be applied in the generation of other clean energy sources. In terms of hydrogen production, the two-dimensional layered type of UT-C_3_N_4_ has more heptazine units, and orderly arranged polar units can provide higher piezoelectricity and improve piezoelectric performance. The piezoelectric photocatalytic performance can reach 12.16 mmol g^−1^h^−1^ relative to ordinary photocatalysis [61], while the UiO-66-NH_2_@CdS composite can reach 2028.45 μmol g^−1^h^−1^ in hydrogen production [70]. It can be seen that piezoelectric photocatalysis can indirectly provide green and clean energy to cope with the energy crisis.

## 4. Application of Piezoelectric Photocatalysis

### 4.1. CO_2_ Reduction

As the large consumption of fossil fuels leads to increasing emissions of carbon dioxide (CO_2_), the CO_2_-induced greenhouse effect seriously affects the environment and human beings. The conversion of CO_2_ into chemical substances or other combustion gasses can alleviate the greenhouse effect and solve certain energy crises. So, it is of great significance to develop technologies and strategies for the conversion of CO_2_ into value-added chemical raw materials using renewable energy sources. Over the past few decades, photocatalytic CO_2_ reduction using light energy has attracted great attention [4,28]. However, due to the problem that photogenerated carriers are easy to compound in photocatalytic CO_2_ reduction, the polarization property of piezoelectric materials can solve this problem via the driving force of the built-in electric field to realize the rapid separation of photogenerated carriers and improve the photocatalytic performance. In 2022, Zhou et al. selected the typical piezoelectric material barium titanate BTO to demonstrate the feasibility of piezoelectric electrocatalytic reduction in CO_2_ [72]. In this work, ultrasonic vibrations were used as the stress acting on BaTiO_3_ to trigger the Piezo-Electrocatalytic Carbon Dioxide Reduction Reaction (PECRR). Yields of up to 63.3 µmol g^−1^ were achieved for the conversion of CO_2_ to CO without the addition of sacrificial agents. BaTiO_3_ produces a very excellent piezoelectric potential, which overcomes the redox potential of CO_2_ and reduces free energy during the vibration. Liu et al. used the pressure generated when a 355 nm pulsed laser was applied to the tetragonal phase BaTiO_3_ (BTO-T) to establish a built-in electric field in BTO-T, which not only modulates the energy band structure for photocatalytic CO_2_RR but also improves the separation of photo-induced carriers to enhance photocatalytic activity [73]. In a closed reactor, the BTO-T had a stable millimolar CO yield (52.9 mmol g^−1^·h^−1^) under pulsed laser irradiation, which was much higher than that under mercury lamp irradiation (0.159 mmol g^−1^·h^−1^), and this is the highest value reported so far for photocatalytic CO_2_RR.

**Figure 10:**
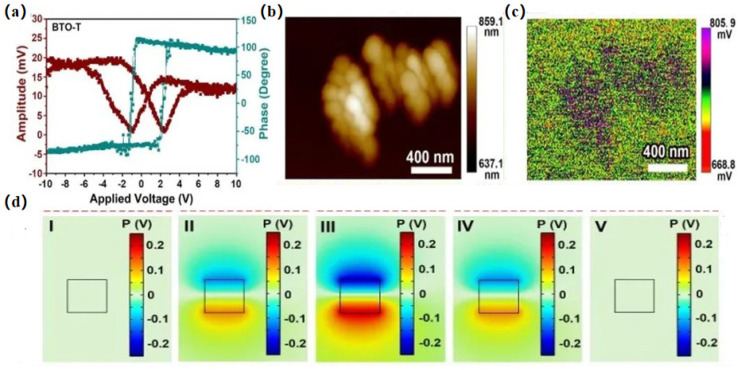
(**a**) Piezoresponsive phase curve and amplitude curve of BTO-T nanoparticles. (**b**) Surface topographic image. (**c**) Surface potential distribution without the 532 nm PL irradiation of BTO-T and (**d**) piezoelectric potential distribution. Reproduced from Ref. [73] with permission. Copyright, 2023. Wiley.

We found that BTO-T has piezoelectric properties from the Piezoresponse Force Microscopy (PFM) diagrams seen in Figure 10a. According to the finite element simulation that simulated the deformation, voltage, and electric field of the BTO-T after being pressurized, this proves that when subjected to pressure, the material is able to produce deformation and a built-in electric field is formed by the pulse, as shown in Figure 10d. The effect of pulsed laser-induced pressure on the surface potential of BTO-T was experimentally demonstrated using Kelvin probe microscopy (KPFM), and the pulsed laser was able to generate a surface potential difference of about 30 mV, as shown in Figure 10b,c.

### 4.2. H_2_O_2_ Evolution

H_2_O_2_ is widely used as an important reactive oxygen species in medical (sterilization, washing, and cell processing), military (incendiary agents), and industrial (foaming agents, bleaching agents, and wastewater treatment) applications. It is also a versatile green oxidant applied in the field of environmental remediation. Despite decades of exploration, the commonly used method for generating H_2_O_2_ is still the anthraquinone method, which has the disadvantages of high energy costs and complexity, and toxic by-pollutant emissions, so the development of new technologies to find the efficient generation of H_2_O_2_ and ROS remains a top priority [74]. The problem with the photocatalytic production of H_2_O_2_ lies in the fact that catalyst materials are unstable when exposed to light, resulting in a shift in their properties, and the conduction bands of many materials are not sufficient to meet the redox potential requirements for the ROS (H_2_O_2_, ·OH) generation reaction. The piezoelectric photocatalytic production of H_2_O_2_ can skillfully circumvent this defect by inducing a built-in electric field in the material through the piezoelectric effect, which effectively inhibits charge complexation, promotes photocatalysis and improves the yield of H_2_O_2_. Bi-based Silleu–Aurivillius materials are layered chalcogenide oxyhalides, which have a wider range of light absorption, better stability, and a relatively positive potential that can effectively serve visible-light-driven water oxidation.

**Figure 11:**
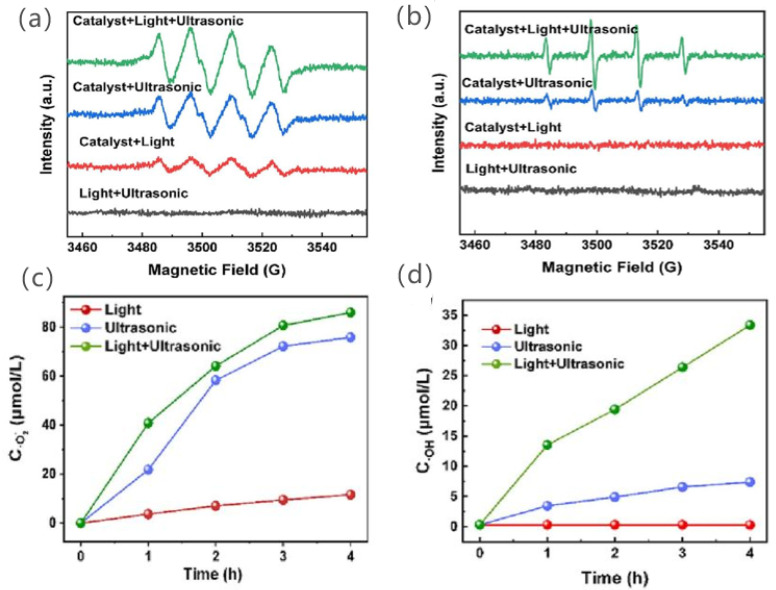
ESR spectra of (**a**) DMPO·O_2_^–^ and (**b**) DMPO·OH with light illumination, ultrasonic treatment, and light-ultrasonic irradiation. Plot of (**c**)·O_2_^–^ and (**d**) OH concentration as a function of the reaction time. Reproduced from Ref. [75] with permission. Copyright, 2023. Elsevier.

Zhang et al. triggered piezoelectric photocatalysis by ultrasonic/visible-light-effective oxygen activation over Bi_4_W_0.5_Ti_0.5_O_8_Cl action with excellent oxygen activation performance, far exceeding that in photocatalysis alone or piezoelectric catalysis [75]. The maximum yield of H_2_O_2_ in the non-sacrificial system with the metal-free deposition of the Bi_4_W_0.5_Ti_0.5_O_8_Cl catalyst reached 530.4 μmol·h^−1^·g^−1^. The catalysts had higher peaks of ·O_2_^−^ and ·OH signals by the ESR spectra of Figure 11a,b under ultrasound and light, indicating higher ·O_2_^−^ and ·OH production. In the tests of piezoelectric catalysis and piezoelectric photocatalysis, the ·O_2_^−^ yields were 19.2 and 22.1 μmol·h^−1^·g^−1^. Hydroxyl radicals were not detected in the photocatalytic process, but the ·OH yield of the piezoelectric photocatalytic system reached 15.8 μmol·h^−1^·g^−1^, which is several times that of the piezoelectric catalytic system alone, as shown in Figure 11c,d. It is also consistent with the previous ESR results.

**Figure 12:**
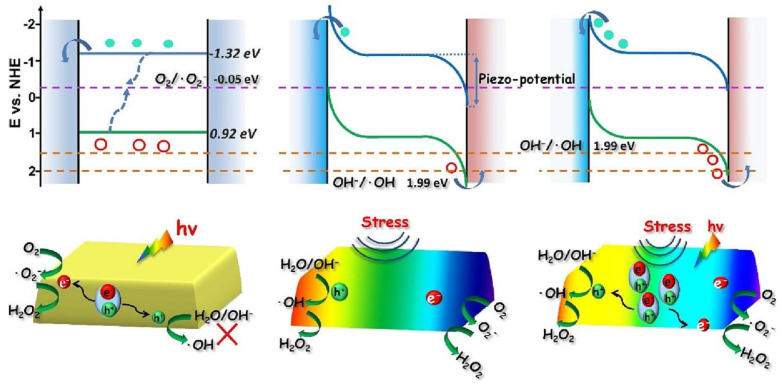
Schematic diagrams of the potential distribution and charge transfer in photocatalysis, piezocatalysis, and piezo-photocatalysis. Reproduced from Ref. [75] with permission. Copyright, 2023. Elsevier.

Figure 12 is a schematic diagram of charge transfer with respect to photocatalysis, piezoelectric catalysis, and piezoelectric photocatalysis, where CB shifts downward with the change in stress in the Mott–Schottky (M-S) plot. Since the piezoelectric potential does not change the bandgap width, the position of VB also moves downward. In piezoelectric catalysis, ultrasonic cavitation produces much larger stress than film bending and generates a sufficiently large positive potential at one end of the material. Under the influence of this bias, the VB at the positive surface moves downward to an extended position below 1.99 eV, bringing the positive charge up to OH/-OH. 

For H_2_O_2_, changing the position of VB/CB in piezoelectric catalysis can produce H_2_O_2_ in both oxygen reduction reaction (ORR) and water oxidation reaction (WOR) pathways. In the case of piezoelectric photocatalysis, the photoexcitation process provides a large amount of free charge, which creates a stronger current of response due to the efficient transfer and separation of charges at the piezoelectric potential and improves the yield of hydrogen peroxide. Zhang et al. investigated the effect of piezoelectricity on the photocatalytic generation of H_2_O_2_ on graphitic carbon nitride (CN) by accurately tuning the molecular structure of graphitic carbon nitride (CN) [76], and the results showed that the role of the piezoelectric effect in the photocatalytic generation of H_2_O_2_ on CN depended largely on the molecular structure of CN. Among them, phosphorus-modified CN (CN-P), oxygen-functionalized CN (CN-OF), and cyano-grafted CN (CN-CA), the photocatalytic activities of CN, CN-P, and CN-OF were increased by about 1.40, 1.46, and 1.51 times, respectively. The introduction of P groups and oxygen groups enhanced the photogenerated charge separation/transfer and piezoelectric polarization and also optimized the active site/reaction potential for the generation of H_2_O_2_ from O_2_, thus synergistically enhancing the piezoelectric effect and ACS catalysis.

### 4.3. H_2_ Evolution

**Figure 13:**
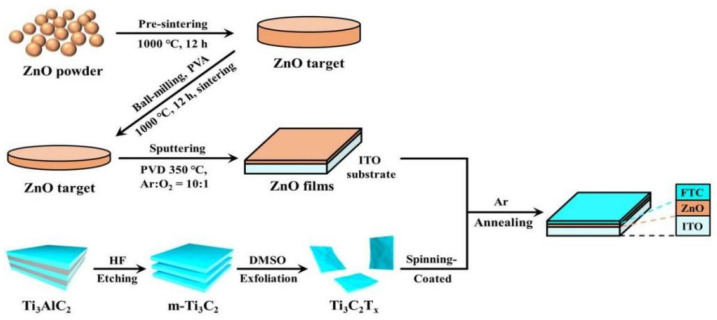
Schematic illustration for the synthetic procedure of ZnO films, FTC, and FTC-ZnO with spinning-coating and annealing. Reproduced from Ref. [79] with permission. Copyright, 2022. Elsevier.

With the continuous development of modern industry, traditional energy resources such as coal, natural gas, and oil are constantly being depleted, and the search for a sustainable clean energy source is an urgent need for every country. Compared to solar, wind, and water energy, which are limited by seasons and geographical locations, hydrogen energy, as a sustainable clean energy source, is of great significance in reducing carbon emissions [77]. Hydrogen energy has come into the limelight due to its own advantages, such as high combustion calorific value, sustainability, abundant reserves, and zero pollution, and its development is capable of realizing a truly green, clean, and sustainable development [78]. Piezoelectric photocatalysis decomposes water into hydrogen by introducing a built-in electric field through the polarization phenomenon to change the physicochemical state and reaction mechanism of the reaction system. This can make up for the shortcomings of photocatalytic water decomposition in high-rate carrier composites and realize efficient hydrogen production from water cracking. Wu et al. constructed Schottky piezoelectric photocatalysts using the metal Ti_3_C_2_T_x_ composite with the inorganic piezoelectric material ZnO, optimized the built-in electric field, overcame the huge potential barrier of the initial ZnO, and improved the piezoelectric photocatalytic hydrogen precipitation performance [79]. Figure 13 illustrates a schematic of the synthesis of the material. 

**Figure 14:**
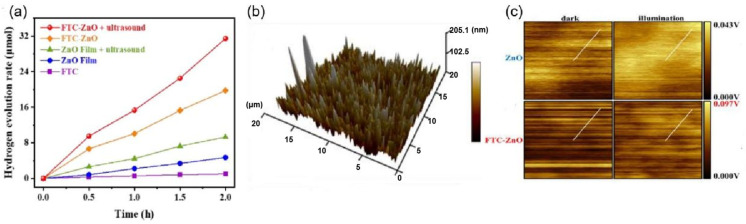
Piezoelectric performance of samples. (**a**) H_2_ yields in FTC-ZnO and ZnO film with/ without ultrasound under Xenon lamp illumination. (**b**) Topography of the height sensor of FTC-ZnO. Their corresponding piezoelectric potential amplitude with the driven bias of 1 V and (**c**) KPFM potential images of ZnO film and FTC-ZnO in the dark and under illumination, corresponding to the measured surface potential. Reproduced from Ref. [79] with permission. Copyright, 2022. Elsevier.

They compared the hydrogen precipitation reactions of ZnO and FTC-ZnO heterojunctions before and after ultrasonic irradiation (all samples were irradiated by a xenon lamp) and found that the H_2_ yield of FTC-ZnO was higher than that of the ZnO film after light and ultrasonic irradiation, as shown in Figure 14a. And Figure 14b shows the morphology and piezoelectric output of the FTC-ZnO at a driving voltage of 1 V. It was found that the FTC-ZnO had a good piezoelectric response. The surface potential range of FTC-ZnO was found to increase to 0~0.097 V by KPFM, which is more than twice that of ZnO. All of the above results demonstrate the superior carrier separation and hydrogen precipitation properties of FTC-ZnO under ultrasound and light, as shown in Figure 14c. 

Cao et al. successfully synthesized ·OH-modified SrTiO_3_ hydroxyl modification by a low-temperature solid-state precursor method, which not only enhances the hydrophilicity and facilitates the contact between the catalyst and water molecules but also generates oxygen defects and accelerates the charge separation and transfer [80]. Thanks to this unique surface functional group modification scheme, the hydrogen production rate of oh-modified SrTiO_3_ under simultaneous irradiation and ultrasonication can reach 701.2 umol h^−1^ g^−1^, which is much higher than that of illumination alone (295.4 umol h^−1^ g^−1^) or vibration (430.8 umol h^−1^ g^−1^). Furthermore, the photocatalytic, piezoelectriccatalytic, and piezoelectric-photocatalytic hydrogen evolution rates of ·OH-modified SrTiO_3_ are all about two times higher than those of pristine SrTiO_3_. Ultrasound-assisted piezoelectric photocatalytic water cracking to hydrogen is still in the early stages of research, and there is still a long way to go to realize practical applications 

### 4.4. Degradation of Contaminants 

**Figure 15:**
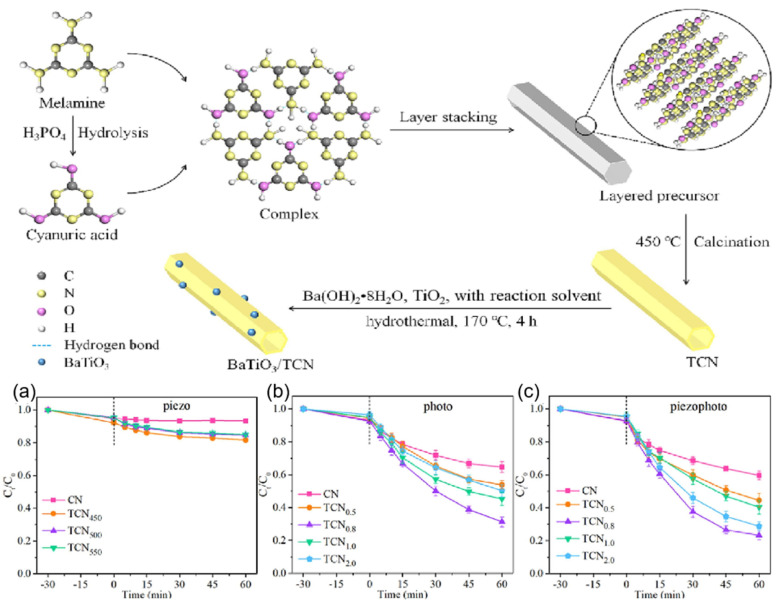
The preparation diagram of the BaTiO_3_/TCN composite. (**a**) Piezoelectric catalytic activity. (**b**) Photocatalytic activity. (**c**) Piezophotocatalytic activity. Reproduced from Ref. [81] with permission. Copyright, 2023. Elsevier.

With the rapid development of industrialization and modernization, the ensuing environmental problems are serious challenges to society. The organic pollutants present in wastewater always affect human health and the environment. Among the existing strategies for treating wastewater pollution, solar-powered photocatalysis hold considerable promise as it is environmentally benign and cost-effective. However, the high complexity of photogenerated carriers severely hampers the development of this technology. Converting organic pollutants into value-added chemicals for low-carbon wastewater discharge remains a major challenge. Piezoelectric catalysis exploits the potential of mechanical energy (e.g., friction, vibration, wind, and renewable energy sources such as tides) [39,40,41,42] for the production of clean energy and water treatment. The generation of electron holes by light and mechanical vibration and the formation of a built-in electric field inside the catalyst promote the effective separation of charge carriers to participate in redox reactions, generating powerful reactive species (ROS) for pollutant removal. Piezoelectric photocatalysis has also been widely reported and studied as an emerging technology for pollutant removal. One-dimensional tubular carbon nitride (TCN) with strong piezoelectricity was synthesized by the hydrothermal method by Huang et al. [81]. The one-dimensional tubular carbon nitride TCN with a non-centrosymmetric structure was combined with classical piezoelectric material, barium titanate, to construct a dual piezoelectric material to maximize the heterojunction and piezoelectric properties on carrier separation to degrade tetracycline. Figure 14 shows a schematic diagram of the synthesis steps and performance testing of experiments. The samples were tested for pure photocatalytic, piezoelectriccatalytic, and piezoelectric-photocatalytic activities for the degradation of tetracycline, respectively. The activity of TCN was found to be significantly higher than that of CN, which is due to the fact that the one-dimensional tubular morphology is more prone to larger deformation under ultrasonic vibration. We can also see that BaTiO_3_/TCN has the best activity for the degradation of tetracycline in piezoelectric photocatalysis, which is due to the polarized electric field generated by ultrasonic activation that promotes the ordered migration of carriers, leading to a higher photoelectric conversion process in the composite material, as shown in Figure 15a–c. 

**Figure 16:**
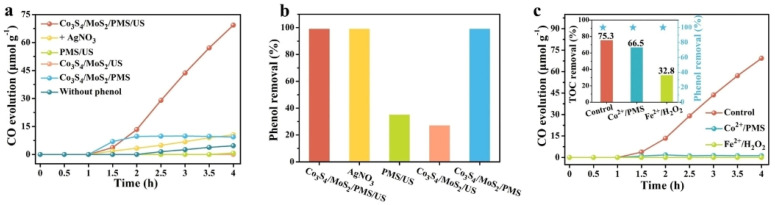
(**a**) CO evolution under different conditions and (**b**) the corresponding removal efficiency of phenol. (**c**) CO evolution in different systems (the inset shows the degradation and TOC removal of phenol); Control stands for the Co_3_S_4_/MoS_2_/PMS/US system. Reproduced from Ref. [82] with permission. Copyright, 2023. Wiley.

Xing et al. prepared a Co_3_S_4_/MoS_2_ catalyst with piezoelectric effect by simple hydrothermal synthesis, and then constructed a coupled system of advanced oxidation and piezoelectric catalysis (Co_3_S_4_/MoS_2_/PMS/US) by introducing peroxymonosulfate (PMS) [82]. The coupled system could degrade 40 ppm phenol to produce 40.72 µmol g^−1^ h^−1^ of CO under sonication with a high selectivity of 74.5%. By prolonging the sonication time, the conversion of phenol to CO could be achieved by up to 16.6%. As shown in Figure 16a–c, this work not only successfully degraded phenol with a degradation rate of 99.9% but also converted phenol into CO during the degradation process, with a corresponding CO yield of 69.32 μmol g^−1^.

## 5. Conclusions and Outlook

In recent years, piezoelectric photocatalysis, as an emerging environmentally friendly technology, has demonstrated great potential in various fields, such as environmental remediation and energy conversion, by triggering reactions through mechanical energy. In this comprehensive review of the principle of the piezoelectric effect, we systematically elucidated the theory that piezoelectric photocatalysts form a built-in electric field through the deformation generated by external stresses during the photocatalytic process, which promotes carrier separation and improves catalytic efficiency. Different types of piezoelectric photocatalysts are also introduced, which play important roles in the fields of environmental remediation and energy conversion and even open up new directions and strategies. Further research and studies are also needed on the physical and chemical properties of piezoelectric photocatalysts, crystal structures, and how to introduce built-in electric fields. From the above review, we can draw a phenomenon that the current work on piezoelectric photocatalysis commonly adopts ultrasonic vibration to generate piezoelectric potentials while there is still a gap in the research on the use of thermal stress, mechanical vibration, air friction and other methods to generate deformation. Moreover, through the above studies, we found that researchers commonly adopt high-frequency ultrasound to generate piezoelectric effect because the higher ultrasound frequency can generate higher piezoelectric potential, which further enhances the catalytic rate. However, the method of low-frequency ultrasound triggering the piezoelectric effect still needs to be explored. The two mechanistic studies derived from piezoelectric photocatalysis are the energy band bending theory and the frequent release and adsorption of polarized surface screening charges, both of which need to be further explored. For example, whether the energy band bending produces a change in the energy levels inside the piezoelectric material or reorganizes the arrangement of electrons, etc., is unclear. More characterization techniques to study the charge conditions in the piezoelectric photocatalytic process are desired.

We have also briefly summarized that, in recent years, piezoelectric photocatalytic technology has been widely used in CO_2_ reduction, H_2_O_2_ generation, the degradation of organic pollutants, wastewater purification, hydrogen production, etc. The built-in electric field generated by the piezoelectric photocatalyst material during deformation is able to make up for the disadvantage of photocatalytic applications in these fields with too high carrier complexes. However, it is not difficult to see that most of the above applications are in liquid-phase devices because ultrasonic vibration and mechanical vibration need the presence of the solvent to be realized. But there are many other ways to cause the deformation of piezoelectric materials, so we need to expand the applications of piezoelectric photocatalyst materials in other aspects. The study on gas-solid phase piezoelectric photocatalysis has not been reported extensively, so the application of piezoelectric photocatalysis to the removal of air pollutants will be a potential field for piezoelectric photocatalysis.

Since piezoelectric photocatalysis is an emerging technology, all the reported investigations are still in the stage of theoretical research and laboratory research. Although some small-scale practical experiments have been explored, the commercial industry application has not been realized yet. We believe it will be realized in the future. There are certainly some challenges and difficulties in their future practical application. First of all, catalysts are very likely to be affected by actual environmental factors, such as light exposure, rainwater washing, and temperature fluctuations, which may lead to performance degradation or structural damage. Thus, both activity and stability in various actual conditions should be considered. For the modification design of piezoelectric photocatalysts, there is mainly elemental doping, the construction of heterojunctions, and the introduction of defect strategies. Composite piezoelectric photocatalysts such as combining the piezoelectric catalyst with piezoelectric photocatalyst and combining the photocatalyst with piezoelectric catalyst appeared in the above reports. However, what kind of roles they played after introducing new heterojunctions or doping also need to be further explored. 

In addition, the kinetics and mechanism of the piezoelectric photocatalytic reaction have not been studied deeply enough, and the key steps such as charge transfer, separation, and complexation in the reaction process, as well as the effects of these steps on the catalytic performance, need to be further explored. Moreover, the device system for studying piezoelectric photocatalytic reactions in the laboratory is a miniature model relative to the real world. In practical applications, where large quantities of gas and water need to be processed, the design and construction of large-scale, highly efficient photocatalytic reaction units is also challenging. Therefore, many problems may arise in practical applications, such as devices that do not fully utilize the piezoelectric effect or inefficient conversion of mechanical energy to electrical energy. All these factors need to be considered in the design of piezoelectric photocatalysts and piezoelectric photocatalytic devices so that it is more closely related to people’s practical applications and in line with a real-life context!

Finally, compared with traditional photocatalytic technology, piezoelectric photocatalytic technology further enhances overall efficiency by generating a built-in electric field through the unique piezoelectricity of the material itself to make up for the shortcoming of the high electron-hole complexation rate in the photocatalytic process. Although piezoelectric photocatalysis has potential in some fields, such as environmental pollution control and new energy development, it is still in the research and exploration stage and has not been widely verified in practical applications. Moreover, piezoelectric photocatalysis also has higher requirements for light and power sources, which require more precise control and regulation. Currently, xenon lamps, as well as high-power ultrasound, are commonly used in piezoelectric catalytic devices, which increases the investment cost a lot and does not utilize the wide-scale popularization of the application. Therefore, the search for triggering the piezoelectric effect through natural mechanical energy, such as wind, water flow, tides, etc., may be more cost-effective and environmentally friendly and will be the focus of designing piezoelectric photocatalysts in the future.

## Figures and Tables

**Table 1:** Comparison of piezoelectric photocatalysts in the reported the literatures.

Catalyst	Reaction Condition	Application Aspects	Catalytic Activity	Ref.
ZnO NRs	Ultrasound (150 W 40 kHz)	Degradation of AO7	80.8% in 100 min	[52]
ZnO-Cl	Ultrasound (100 w 40 kHz)	Degradation of RhB	75% in 50 min	[54]
BTO-OV2	UItrasound (40 kHz	N_2_ reduction	106.7 μmol g^−1^h^−1^	[23]
Ba_0.85_Sr_0.15_TiO_3_	UItrasound (0.81 W/cm^2^, 40 kHz)	Degradation of RhB	9292.6% in 16 min	[58]
UT-g-C_3_N_4_	Ultrasound (240 W, 40 kHz)	H_2_ evolution	12.16 mmol g^−1^h^−1^	[61]
CNPC	UUltrasound (200 W, 40 kHz)	Degradation of atrazine	94% in 60 min	[45]
PVDF-TrFE-BiFeO_3_	Ultrasound (100 w, 45 kHz)	Degradation of RhB	100% in 20 min	[65]
UiO-66-NH_2_@CdS	Ultrasound (150 W, 40 kHz)	H_2_ evolution	2028.45 μmol g^−1^h^−1^	[70]
